# Influence of Age and Geography on Chemical Composition of 98043 Urinary Stones from the USA

**DOI:** 10.1016/j.euros.2021.09.011

**Published:** 2021-10-28

**Authors:** Jonathan E. Katz, Nachiketh Soodana-Prakash, Anika Jain, Madhumita Parmar, Nicholas Smith, Oleksandr Kryvenko, Glenn W. Austin, Hemendra N. Shah

**Affiliations:** a Department of Urology, Miller School of Medicine, University of Miami, Coral Gables, FL, USA; b Department of Pathology, Miller School of Medicine, University of Miami, Coral Gables, FL, USA; c Louis C. Herring Laboratory, Orlando, FL, USA

**Keywords:** Urolithiasis, Epidemiology, Calcium oxalate stone, Uric acid stones, stone composition, calcium phosphate stone, struvite stone, stone analysis

## Abstract

**Background:**

Urolithiasis is a growing issue globally, but it is heterogeneous, with a different epidemiology and pathophysiology for each different stone composition.

**Objective:**

The purpose of this study is to describe the incidence of urinary stones in the USA from 2016 to 2019 by chemical composition and to investigate the influence of age and geography on these stone types.

**Design, setting, and participants:**

We obtained compositional analyses for all urinary stones submitted to a national laboratory over an approximately 3-yr period.

**Outcome measurements and statistical analysis:**

Data collected included the chemical constituents of a stone, patient age, and geographical origin. We describe the incidence of each stone type by frequency. Statistical testing was performed to determine the influence of age and geographical region on overall incidence of each stone composition.

**Results and limitations:**

In total, 99 908 specimens were analyzed. When pure stones were ordered by frequency, we found that the most common stone type was calcium oxalate (CaOx) (79.2%), followed by uric acid (UA; 14.3%), calcium phosphate (CaPO_4_; 3.7%), cystine (0.51%), drug induced (0.12%), and magnesium ammonium phosphate (0.04%). CaOx, UA, and CaPO_4_ were often mixed with one another. Among CaOx stones, the plurality (28.0%) was made of pure calcium oxalate monohydrate (COM), and only 0.002% was pure calcium oxalate dihydrate. There was an overall association between stone composition and both geographical distribution and age (*p* < 0.001).

**Conclusions:**

CaOx stones comprise the majority of urinary stones in the USA, of which almost 28% were pure COM. Additionally, age and geographical region are significantly associated with variations in stone composition.

**Patient summary:**

We evaluated the incidence of urinary stones in the USA based on their chemical composition. The most common stone type was calcium oxalate, the majority of which was pure calcium oxalate monohydrate. We also found age and geographical region to be significantly associated with variations in stone composition.

## Take Home Message

In this report, we examined the incidence of urinary stones in the USA and categorized their frequency based on chemical composition. We found that calcium oxalate made up ~80% of overall stones, of which almost 50% were pure calcium oxalate monohydrate. We also found that age and geographical region were significantly associated with variations in stone composition.

## 1 Introduction

Urolithiasis is a growing issue globally, and the prevalence of stone disease continues to rise in the USA [1]. Data from the National Health and Nutrition Examination Survey from 2007 to 2010 demonstrated that the prevalence of kidney stones was 10.6% in males and 7.1% in females [2]. However, urolithiasis is also a heterogeneous condition with a different epidemiology and pathophysiology corresponding to the different stone compositions, which are influenced by multiple factors including age and geography [3], [4], [5].

For example, calcium oxalate (CaOx) stones comprise the plurality of urinary stones [6], [7]; however, internationally, the prevalence of CaOx stones varies over a wide range, comprising anywhere from 44.7% of stones in Oman [6] to 84% of stones in Germany [7]. Additionally, several studies have investigated geographical variation in stone composition in individual countries [7], [8], [9]. The most systematic USA-based study that investigated regional variation in stone composition examined 4335 stones originating from seven representative states and concluded that the only significant geographical variation in stone composition was an increase in uric acid (UA) stones in Florida [10].

Understanding the epidemiology of each urinary stone type, including which form in isolation and which crystalize together, as well as the influence of aging and geography on the incidence of each stone type is important because it both helps quantify the disease burden for each specific stone type and provides insights into the underlying pathophysiologic processes related to stone formation [11], [12]. Therefore, using data submitted to a high-volume urinary stone analysis laboratory in the USA, we characterized the different frequencies with which stones of different chemical compositions form in isolation and in what combinations they form together. Furthermore, we investigated the influence of age and geography on the frequency of these different stone types.

## 2 Materials and methods

The study was exempt from our institutional review board approval as no identifiable personal health information was collected or analyzed. We obtained compositional analyses for all urinary stones submitted to the Louis C. Herring Laboratory (Orlando, FL) over a period of approximately 3 yr, from July 15, 2016 to September 29, 2019. The laboratory receives stone samples from the entire country. The stones were submitted from diverse settings, including community and academic hospitals, clinics, and reference laboratories. Data collected included the chemical composition of each stone, patient’s age, and geographical location from which the stone was sent.

Stone composition was determined by integrative crystallography, optical and chemical microscopy supplemented with infrared spectrophotometry, and/or x-ray diffractometry as indicated. The same methods were used to identify nonurinary stones. As most stones contained multiple chemical components, the analysis included detailed chemical composition and percent contribution of up to eight chemical components [13].

For the analysis of the effect of age and geography on overall incidence of each stone type, stones were classified based on their single largest component. Additionally, unless urinary stones were referred to as pure, they were classified by the largest component (eg, a stone found to contain 40% calcium oxalate monohydrate [COM], 30% UA, and 30% calcium phosphate [CaPO_4_] would be classified as a CaOx stone). Of note, uric acid anhydrous (UAA) and dihydrate stones were grouped together as UAA stones because dihydrate is less stable and known to transform into UAA, which is more thermodynamically stable [14]. Stones unlikely to have their origin in the urinary tract were classified as artifacts.

To eliminate random variation due to state boundaries, we pooled the states into seven regions according to American Urological Association subsections: New England, New York, Mid-Atlantic, North Central, Southeastern, South Central, and Western. Age was categorized by decade.

### 2.1 Statistical analysis

Descriptive statistics regarding the age of patients and incidence of each type of stone were calculated. For categorical variables, we reported frequency and percentages, and for continuous variables, we reported mean, standard deviation, and median. We performed chi-square testing to determine whether there was an influence of age and geography on the incidence of the different stone types. This was repeated for subtypes of pure CaOx (COM, calcium oxalate dihydrate [COD], and mixed CaOx stone), subtypes of pure UA stones (UAA, ammonium urate, and sodium urate), and mixed CaOx and UA stones. All statistical analyses were conducted using STATA v16.1.

## 3 Results

In total, 99 908 specimens were sent for kidney stone analysis between July 15, 2016 and September 29, 2019, of which 98 043 were categorized as urinary stones. Details of age and geography of the specimens were available for 99 004 and 89 588 stones, respectively. The mean age of patients was 56.49 ± 16.84 yr (range 1–99 yr, median 58 yr with an interquartile range of 45–69 yr), and stone specimens from all geographical regions were available for analysis. When pure stones were ordered by frequency, we found that the most common stone type was CaOx (79.2%), followed by UA (14.3%), CaPO_4_ (3.7%), cystine (0.51%), drug induced (0.12%), and magnesium ammonium phosphate (MAP, 0.04%; Table 1). Findings were similar for stones classified by the largest component: CaOx (77.6%), UA (10.9%), CaPO_4_ (7.2%), MAP (2.4%), cystine (0.23%), and drug induced (0.08%; Table 1).

**Table 1:** Urinary stones classified by pure or predominant chemical composition: frequency, age, and distribution

Stone composition	Predominant stone component			Pure stone composition		
	Number of stones	%	Mean age ± SD (median)	Number of stones	%	Mean age ± SD (median)
CaOx	69 852	77.6	55.87 ± 16.53 (58)	30 729	79.19	59.27 ± 15.17 (61)
UA	9784	10.9	64.22 ± 13.07 (66)	5549	14.3	63.41 ± 13.17 (65)
CaPO_4_	6419	7.1	52.19 ± 19.43 (53)	1439	3.7	50.92 ± ± 19.94 (53)
MAP	2165	2.4	58.38 ± 19.68 (61)	17	0.04	50.07 ± 21.96 (51)
Cystine	231	0.26	42.14 ± 19.85 (40)	199	0.51	42.17 ± 20.10 (40)
Artifacts	1394	1.6	53.44 ± 19.68 (56)	809	2	54.40 ± 19.82 (58)
Drugs	74	0.082	56.44 ± 17.58 (59)	48	0.12	55.52 ± 19.23 (59)
Other	13	0.014	43.07 ± 22.52 (41)	13	0.03	43.07 ± 22.52 (41)
Equal amount of two components	347	0.39	53.27 ± 18.25 (53)	–	–	–
Total	90 004	100		38 803	100	

CaOx = calcium oxalate; CaPO_4_ = calcium phosphate; MAP = magnesium ammonium phosphate; SD = standard deviation; UA = uric acid.

**Fig. 1:**
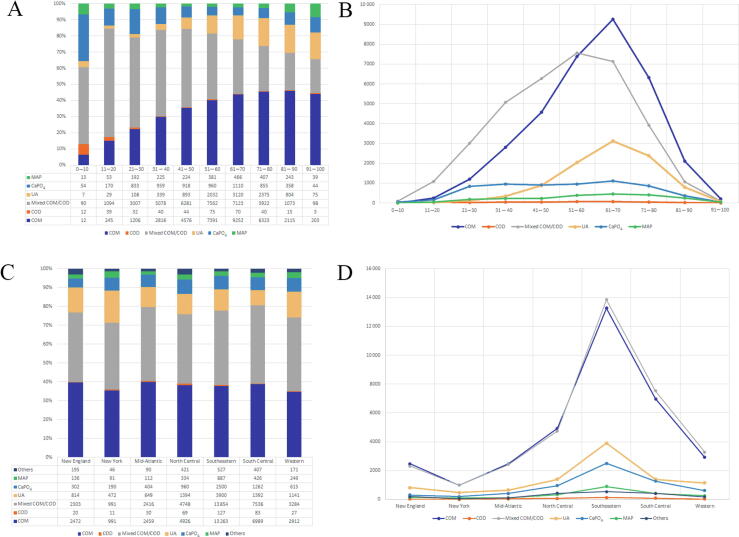
Chemical composition of urinary stones classified by age: (A) percentile and (B) frequency. Chemical composition of urinary stones classified by region: (C) percentile and (D) frequency. CaPO_4_ = calcium phosphate; COD = calcium oxalate dehydrate; COM = calcium oxalate monohydrate; MAP = magnesium ammonium phosphate; UA = uric acid.

Regarding variation of stone type with age, the incidence of COM and UA stones increased with age and that of CaPO_4_ decreased with age (*p* < 0.001). MAP stones had a bimodal distribution, making up a larger percentage of overall stones in the first and last decades of life (*p* < 0.001; Fig. 1A and 1B). Similarly, there was an overall association between stone composition and geographical distribution (*p* < 0.001). New York had the highest incidence of UA stones and a lower COM and mixed CaOx stone burden, while New England and the Mid-Atlantic had the highest COM burden (Fig. 1C and 1D).

**Fig. 2:**
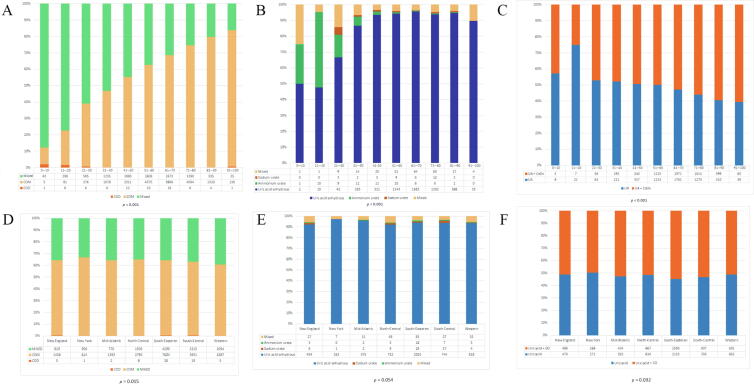
Impact of age by decade on (A) pure CaOx stones, (B) pure UA stones, and (C) combined CaOx and UA stones. Impact of geography by AUA region on (D) pure CaOx stones, (E) pure UA stones, and (F) combined CaOx and UA stones. AUA = American Urological Association; CaOx = calcium oxalate; CO = calcium oxalate; COD = calcium oxalate dehydrate; COM = calcium oxalate monohydrate; UA = uric acid.

Among specimens with CaOx as the predominant component, 44.0% were pure CaOx (28.0% COM, 0.10% COD, and 15.9% mixed COM and COD). The remaining 56.0% of specimens were mixed most commonly with CaPO_4_ (51.0%), followed by UA (3.4%; Table 2). Additionally, within the combined CaOx and CaPO_4_ stones, hydroxyapatite was the most commonly found subtype of CaPO_4_ stone. Regarding the effect of age on pure CaOx stones, we found that the proportion of pure COD and mixed CaOx stones reduces with age, while that of COM increases with age (*p* < 0.001; Fig. 2A). Regarding the effect of geography, on pure CaOx stones, New York had the highest percentage of COM stones (*p* = 0.015; Fig. 2D).

**Table 2:** Predominant CaOx stones: frequency and age distribution

Stone composition		Number of stones	%	Mean age ± SD (median)
Pure stone composition	COD	71	0.10	51.25 ± 20.27 (52)
	COM	19 576	28.02	61.90 ± 13.95 (63)
	Mixture of COM + COD	11 082	15.86	54.67 ± 16.06 (56)
Predominant stone composition	Majority CaOx + CaPO_4_			
	Hydroxyapatite	16 533	23.67	54.11 ± 16.28 (56)
	Carbonate apatite	95	0.14	55.21 ± 15.61 (56)
	Brushite	26	0.04	64.11 ± 13.67 (65.5)
	Mixed CaPO_4_ subtypes	18 988	27.18	50.51 ± 17.38 (50)
	CaOx + UA	2391	3.42	64.60 ± 13.38 (66)
	CaOx + MAP	4	0.01	50.5 ± 16.21 (50)
	CaOx + one other stone type	98	0.14	62.53 ± 12.07 (64.5)
	CaOx + 2 additional components	904	1.29	60.02 ± 16.97 (62)
	CaOx+ 3 additional components	84	0.12	56.35 ± 19.55 (57)
Total		69 852	100	

CaOx = calcium oxalate; CaPO_4_ = calcium phosphate; COD = calcium oxalate dehydrate; COM = calcium oxalate monohydrate; MAP = magnesium ammonium phosphate; SD = standard deviation; UA = uric acid.

Among specimens with UA as the predominant component, 56.7% were pure UA (53.1% UAA, 0.71% ammonium urate, 0.41% sodium urate, and 2.5% mix of UA subtypes; Table 3). The remaining 43.3% of specimens were mainly mixed with CaOx (39.6%), and very rarely with CaPO_4_ (0.22%) or MAP (0.06%; Table 3). Regarding the influence of age on the incidence of pure UA stones, we found that the proportion of ammonium urate stones decreased with age and that of UAA stones increased with age (*p* < 0.001; Fig. 2B and 2C). Additionally, when comparing the proportion of pure UA stones with that of mixed UA and CaOx stones, the proportion of mixed UA and CaOx stones increases with age (*p* < 0.001; Fig. 2B). No significant differences were found between the subtypes of pure UA stones and geographical origin (Fig. 2C); however, when comparing the proportion of pure UA stones with that of mixed UA and CaOx stones, there was a significant increase in the proportion of combined UA and CaOx stones in the Southeast region (*p* = 0.032; Fig. 2F).

**Table 3:** Predominant UA stones: frequency and age distribution

Stone composition		Number of stones	%	Mean age ± SD (median)
Pure stone composition	UAA and/or dihydrate	5199	53.14	63.72 ± 12.73 (65)
	Ammonium urate	69	0.71	44.84 ± 19.75 (42)
	Sodium urate	40	0.41	61.07 ± 16.64 (64)
	Mixture of UAA + ammonium urate + sodium urate	241	2.46	62.35 ± 16.65 (64)
Predominant stone composition	UA + CaOx			
	UAA + COM	3417	34.92	66.25 ± 12.17 (67)
	UAA + COD	24	0.25	61.25 ± 11.73 (60.5)
	UAA + COM/COD	152	1.55	59.51 ± 11.46 (59.5)
	Ammonium urate + COM	23	0.24	51.30 ± 19.64 (45)
	Ammonium urate + COD	3	0.03	52.33 ± 4.61 (55)
	Ammonium urate + COM/COD	13	0.13	43.15 ± 13.29 (41)
	Sodium urate + COM	4	0.04	40.25 ± 26.08 (32.5)
	Multiple subtypes of UA with CaOx mixtures	234	2.39	64.85 ± 13.36 (67)
	UA + CaPO_4_	22	0.22	55.72 ± 16.67 (56.5)
	UA + MAP	6	0.06	58.5 ± 22.48 (59)
	UA + one other stone	56	0.57	65.57 ± 12.36 (66)
	UA + 2 additional components	251	2.57	60.58 ± 15.96 (63)
	UA + 3 additional components	28	0.29	63.92 ± 14.30 (67.5)
	UA + 4 additional components	2	0.02	52.00 ± 21.21 (52)
Total		9784	100	

CaOx = calcium oxalate; CaPO_4_ = calcium phosphate; COD = calcium oxalate dehydrate; COM = calcium oxalate monohydrate; MAP = magnesium ammonium phosphate; SD = standard deviation; UA = uric acid; UAA = uric acid anhydrous.

Among specimens with CaPO_4_ as the predominant component (Table 4), only 0.92% were a single component, and 22.4% were a combination of different types of CaPO_4_ of which the single largest component was hydroxyapatite (12.1% of CaPO_4_ specimens; Table 5). The remaining CaPO_4_ specimens were mixed commonly with COM (32.7%), COM and COD (15.3%), or MAP (15.4%). When found mixed with UA, CaPO_4_ stones primarily combine with only the ammonium urate subtype of UA stones.

**Table 4:** Predominant CaPO_4_ stones: frequency and age distribution

	Stone composition	Number of stones	%	Mean age ± SD (median)
Pure stone composition	Hydroxyapatite	1	0.02	75
	Carbonate apatite	10	0.16	67.7 ± 14.56 (70.5)
	Brushite	48	0.75	43.81 ± 19.15 (42)
	Combination of CaPO_4_ subtypes			
	Hydroxyapatite with smaller amount of carbonate apatite	774	12.06	49.91 ± 20.64 (51)
	Carbonate apatite with smaller amount of hydroxyapatite	7	0.11	63.28 ± 19.65 (60)
	Brushite and hydroxyapatite	125	1.95	47.96 ± 18.57 (47)
	Brushite and carbonate apatite	3	0.05	37.66 ± 31.34 (43)
	Brushite and whitlockite	67	1.04	65.14 + 17.63 (67)
	Combination of >2 CaPO_4_ subtypes	404	6.29	51.67 ± 18.16 (53.5)
Predominant stone composition	Combination of CaPO_4_ with CaOx			
	CaPO_4_ subtypes + COM			
	Hydroxyapatite	2	0.03	65 ± 5.65 (65)
	Carbonate apatite	9	0.14	60.55 ± 63 (63)
	Brushite	134	2.09	53.36 ± 16.01 (54)
	Multiple CaPO_4_ subtypes	2098	32.68	50.7 ± 19.11 (50)
	CaPO_4_ subtypes + COD			
	Carbonate apatite	1	0.02	81
	Brushite	5	0.08	46.8 ± 17.45 (50)
	Multiple CaPO_4_ subtypes	146	2.27	51.01 ± 21.08
	CaPO4 subtypes + COM + COD			50.91 ± 18.35 (51)
	Hydroxyapatite	2	0.03	38.5 ± 24.74 (38.5)
	Carbonate apatite	4	0.06	70.75 ± 9.28 (68)
	Brushite	24	0.37	51.83 ± 16.93 (56)
	Multiple CaPO_4_ subtypes	979	15.25	50.83 ± 18.37 (50)
	Combination of CaPO4 + UA			
	Carbonate apatite	3	0.05	49 ± 27.18
	Brushite	1	0.02	61
	Multiple CaPO_4_ subtypes	17	0.26	49.81 ± 24.15 (53)
	Combination of CaPO4 + MAP	985	15.35	56.62 + 19.83 (60)
	CaPO_4_ + one other component	4	0.06	46.5 ± 17.25 (45)
	CaPO_4_ + 2 additional components	526	8.19	55.37 + 19.49 (58)
	CaPO_4_ + 3 additional components	40	0.62	56.30 + 16.25 (57.5)
Total		6419	100	

CaOx = calcium oxalate; CaPO_4_ = calcium phosphate; COD = calcium oxalate dehydrate; COM = calcium oxalate monohydrate; MAP = magnesium ammonium phosphate; SD = standard deviation; UA = uric acid.

**Table 5:** Total number of single largest component MAP stones with average age, standard deviation, and median

	Stone composition	Number of stones	%	Mean age ± SD (median)
Pure stone composition	Pure struvite	16	0.73903	52.81 ± 20.83 (52.5)
	Newberyite	1	0.0461894	17
Predominant stone composition	MAP + COD	1	0.0461894	64
	MAP + carbonate apatite	810	37.413395	59.60 ± 19.67 (63)
	MAP + multiple forms of CaPO_4_	64	2.9561201	59.04 ± 19.07 (62)
	MAP + CaPO_4_ + UA			
	Ammonium urate	523	24.157044	56.84 + 20.30 (60)
	Ammonium urate with other uric acid subtypes	3	0.1385681	57.12 + 20.18 (60)
	MAP + any CaPO_4_ + COD	29	1.3394919	61.13 + 20.81 (67)
	MAP + any CaPO_4_ + COM	312	14.411085	60.31 + 19.06 (64)
	MAP + any CaPO_4_ + mixed CaOx	171	7.8983834	59.24 + 14.84 (54.5)
	MAP + any CaPO_4_ + drug stone	2	0.0923788	54.5 + 14.84 (54.5)
	MAP combined with 3 additional components	233	10.762125	52 + 19.48 (56)
Total		2165	100	

CaOx = calcium oxalate; CaPO_4_ = calcium phosphate; COD = calcium oxalate dehydrate; COM = calcium oxalate monohydrate; MAP = magnesium ammonium phosphate; SD = standard deviation; UA = uric acid.

Among specimens with MAP as the predominant component, only 0.08% were a single component. The majority of specimens were combined with carbonate apatite (34.7%). Additionally, they commonly combine with CaPO_4_ and either ammonium urate (24.2%) or COM (14.4%; Table 5). Similar to CaPO_4_ predominant stones, MAP predominant stones primarily combine with only the ammonium urate subtype of UA stones.

Of the cystine stones, 86% were found to be pure and had the lowest average age among all stone types (42.14 ± 19.85 yr).

## 4 Discussion

Overall, similar to the data from a large German cohort, CaOx stones are the most common type of urinary stone (79.2%), followed by UA stones (14.3%) [7]. When we classified the incidence of both pure and mixed stones, we found only small changes in overall percentile, which justified our decision to analyze the effect of age and gender on the frequency of stone type classified based on predominant component.

As individuals age, the overall prevalence of urinary stones appears to increase linearly until it peaks at the age of 60–70 yr [7]. Regarding age and the shifting composition of various urinary stone types, prior research in the USA demonstrated that CaPO_4_ stones decreased with age, UA stones increased with age [15], [16], and CaOx stones were more common in older individuals [16]. Internationally, COD stones decreased with age in both an Israeli [17] and a French cohort [18]. Similarly, we found that UA and CaOx increased with age, while COD frequency peaks in the first decade of life and makes up <1% of urinary stones once individuals reach ≥20 yr of age. Reasons for this may be explained by both metabolic differences between the age groups and the stability of the different crystalline structures.

Regarding the clinical differences believed to favor the relative ratio of COD/COM, hyperoxaluric states appear to promote COM, while COD is more common in hypercalcemic states [19]. A decline in the prevalence of COD stones with age may be related to a reduction in urinary calcium excretion with age [19]. Additionally, COM crystals are more chemically stable [20], and it is possible that even though COD forms initially, it transforms to COM over time.

Regarding geographical variation in the USA, in one small study, there was an increase in UA stones in Florida [10]. In Germany, UA stones had the highest frequency in the eastern and southern regions [7]. Similarly, in Turkey, UA stones were most common in the Eastern Anatolia region where animal protein consumption is thought to be higher [8]. In our cohort, New York had the highest UA stone burden, and New England and the Mid-Atlantic regions had the highest COM burden. However, the overall differences were relatively small, and many factors including age, overall meat intake, rates of hyperuricosuria, and rates of acidic urine could be responsible for these differences.

We also analyzed which other stone types were most likely to combine with CaOx and found that CaPO_4_ (most commonly hydroxyapatite) stones accounted for 91.1% of mixed CaOx stones. The frequent crystallization of CaOx with CaPO_4_ is consistent with the hypothesis of Randall’s plaques as a precursor lesion, where initial deposition of hydroxyapatite acts as a nidus for further deposition of CaOx crystals [21].

We analyzed the influence of age and geography on the subtypes of UA stones. Both the overall incidence of pure UA stones and the incidence of combined UA and CaOx stones increase with age, although the latter increases at a greater rate. In view of the well-established pH dependence of UA stones, the rising proportion of UA stones with age might be attributable to the progressive defects in urine ammonia excretion that manifests in decreasing the urinary pH [22].

We also analyzed which other stone types were likely to mix with UA stones, and we found that 80.6% of mixed UA stones were primarily mixed with COM. These combined stones also compromised a larger proportion of the overall stone burden as individuals age. Presumably, the same physiologic factors that increase the incidence of COM and UA individually also increase their combined incidence.

Of UA subtypes, pure UA (UAA with or without dihydrate) was the most abundant subtype at all ages; however, the incidences of ammonium urate and sodium urate stones were more common in the younger age group. Of note, both ammonium urate stones and metabolic diseases are more common in younger patients. These patients often have chronic diarrhea, which causes a high urinary level of ammonium urate and formation of these stones [23].

CaPO_4_ stones rarely form as a single subtype (<1%) and are much more frequently found in combination with one another (∼20%) of which the single largest component is often hydroxylapatite. These stones are also commonly mixed with CaOx or MAP. Owing to the small number of pure CaPO_4_ stones, we did not analyze the effect of age or geographical region on their incidence of these stones; however, it was noted that pure brushite stones occurred in younger patients (43.81 ± 19.15 yr old). It is important to identify patients with brushite stones because on metabolic evaluation, they are at increased risk for recurrence and all have a metabolic abnormality, most commonly hypercalciuria or elevated urinary pH [24].

Most MAP stones were found in combination with carbonate apatite or multiple forms of CaPO_4_. Mixed MAP and UA stones are found only in combination with ammonium urate. This relationship is likely secondary to both stones forming in alkaline urine in the setting of urinary tract infections with urease-producing organisms [23], [25].

Although we believe that this is the largest cohort of stones from the USA to be investigated, there are several limitations to this study. This study includes data for all urinary stones, including lower urinary tract stones. However, the incidence of lower urinary tract stones has been decreasing and likely represents ≤5% of the sample [26]. Additionally, because our stone cohort is taken from stones submitted for chemical analysis, this disproportionately represents stones requiring intervention. With that said, having been brought to medical attention or requiring surgical intervention, these stones are the most clinically significant urinary stones and therefore the stones most important to focus on for epidemiologic purposes. Finally, this study used cross-sectional data from the period of July 15, 2016 to September 29, 2019 sent to one high-volume chemical analysis laboratory located in Florida. This may bias our sample to the Southeast region; therefore, we did not attempt to compare the overall prevalence of urinary stones in the different regions of the USA. Another limitation includes the lack of clinical data available for each specimen such as the relevant patient’s gender, race, past medical history, or comorbidities.

## 5 Conclusions

This series presents the largest analysis to date of urinary stone composition in the USA. Age and geographical region were significantly associated with variations in stone composition, with CaOx making up ∼80% of overall stones, of which almost 50% were pure COM. Further research into the chemistry and metabolic derangements underlying the COD/COM ratio and combination of CaOx and UA stones is necessary for the development of potential targets for the prevention and treatment of these highly prevalent stones.

  ***Author contributions*:** Hemendra N. Shah had full access to all the data in the study and takes responsibility for the integrity of the data and the accuracy of the data analysis.

*Study concept and design*: Katz, Shah, Smith, Austin.

*Acquisition of data*: Kryvenko, Austin.

*Analysis and interpretation of data*: Soodana-Prakash, Jain, Parmar.

*Drafting of the manuscript*: Katz, Jain.

*Critical revision of the manuscript for important intellectual content*: Austin, Shah, Smith, Kryvenko.

*Statistical analysis*: Soodana-Prakash, Jain, Parmar.

*Obtaining funding*: None.

*Administrative, technical, or material support*: Soodana-Prakash.

*Supervision*: Shah.

*Other*: None.

  ***Financial disclosures:*** Hemendra N. Shah certifies that all conflicts of interest, including specific financial interests and relationships and affiliations relevant to the subject matter or materials discussed in the manuscript (eg, employment/affiliation, grants or funding, consultancies, honoraria, stock ownership or options, expert testimony, royalties, or patents filed, received, or pending), are the following: None.

  ***Funding/Support and role of the sponsor*:** None.
