# Supplementary material for: Influence of Age and Geography on Chemical Composition of 98043 Urinary Stones from the USA
Source: Eur Urol Open Sci. 2021 Oct 28;34:19–26. doi: 10.1016/j.euros.2021.09.011 (PMC8655393; doi:10.1016/j.euros.2021.09.011)
Supplement: Supplementary data 1 [file mmc1.docx]

Supplementary table 1- Total Number of Single Largest Component MAP Stones with Average Age, Standard Deviation, and Median

|  | **Stone Composition** | **Number of Stones** | **%** | **Mean Age±SD (Median)** |
| --- | --- | --- | --- | --- |
| **Pure Stone Composition** | Pure Struvite | 16 | 0.73903 | 52.81 ±20.83(52.5) |
|  | Newberyite | 1 | 0.0461894 | 17 |
| **Predominant Stone Composition** | MAP + COD | 1 | 0.0461894 | 64 |
|  | MAP + Carbonate Apatite | 810 | 37.413395 | 59.60±19.67(63) |
|  | MAP + Multiple forms of CaPO4 | 64 | 2.9561201 | 59.04±19.07(62) |
|  | MAPs + CaPO4 + UA | | | |
|  | Ammonium Urate | 523 | 24.157044 | 56.84 + 20.30 (60) |
|  | Ammonium Urate with other uric acid subtypes | 3 | 0.1385681 | 57.12 + 20.18 (60) |
|  | MAP + any CaPO4 + COD | 29 | 1.3394919 | 61.13 + 20.81 (67) |
|  | MAP + any CaPO4 + COM | 312 | 14.411085 | 60.31 + 19.06 (64) |
|  | MAP + any CaPO4 + mixed CaOx | 171 | 7.8983834 | 59.24 + 14.84 (54.5) |
|  | MAP + any CaPO4 + drug stone | 2 | 0.0923788 | 54.5 + 14.84 (54.5) |
|  | MAP combined with 3 additional components | 233 | 10.762125 | 52 + 19.48 (56) |
| **Total** |  | **2165** | **100%** |  |
